# The clinical outcomes of COVID-19 critically ill patients co-infected with other respiratory viruses: a multicenter, cohort study

**DOI:** 10.1186/s12879-023-08010-8

**Published:** 2023-02-06

**Authors:** Khalid Al Sulaiman, Ohoud Aljuhani, Hisham A. Badreldin, Ghazwa B. Korayem, Abeer A. Alenazi, Ahlam H. Alharbi, Albandari Alghamdi, Alaa Alhubaishi, Ali F. Altebainawi, Mohammad Bosaeed, Rand Alotaibi, Ahad Alawad, Nirvana Alnajjar, Khalid Bin Saleh, Walaa A. Sait, Samiah Alsohimi, Meshari M. Alanizy, Sarah A. Almuqbil, Ibrahim Al Sulaihim, Ramesh Vishwakarma, Mai Alalawi, Fatimah Alhassan, Suliman Alghnam

**Affiliations:** 1 Pharmaceutical Care Department, King Abdulaziz Medical City (KAMC)-Ministry of National Guard Health Affairs (MNGHA), Riyadh, Saudi Arabia; 2 College of Pharmacy, King Saud bin Abdulaziz University for Health Sciences, PO Box 22490, Riyadh, 11426 Saudi Arabia; 3 King Abdullah International Medical Research Center, Riyadh, Saudi Arabia; 4 Norwich Medical School, University of East Anglia, Norwich, UK; 5 Department of Pharmacy Practice, Faculty of Pharmacy, King Abdulaziz University, Jeddah, Saudi Arabia; 6 Pharmaceutical Care Services, King Khalid Hospital, Hail Health Cluster, Hail, Saudi Arabia; 7 Department of Pharmacy Practice, College of Pharmacy, Princess Nourah bint Abdulrahman University, P.O.Box 84428, Riyadh, 11671 Saudi Arabia; 8 Pharmaceutical Care Department, Prince Sultan Military Medical City, Riyadh, Saudi Arabia; 9 Department of Pharmaceutical Sciences, Fakeeh College for Medical Sciences, Jeddah, Saudi Arabia; 10 Population Health Section, King Abdullah International Medical Research Center, Riyadh, Saudi Arabia; 11 Infectious Disease Care Department, King Abdulaziz Medical City, Riyadh, Saudi Arabia; 12 College of Medicine, King Saud bin Abdulaziz University for Health Sciences, Riyadh, Saudi Arabia; 13 College of Pharmacy, Qassim University, Qassim, Saudi Arabia; 14 Pharmaceutical Care Department, King Abdulaziz Medical City, Jeddah, Saudi Arabia; 15 Pharmaceutical Care Department, King Abdulaziz University Hospital, Jeddah, Saudi Arabia; 16 Central Security Hospital, Riyadh, Saudi Arabia; 17 Pharmaceutical Care Department, King Fahad Armed Forces Hospital, Jeddah, Saudi Arabia; 18 Saudi Critical Care Pharmacy Research (SCAPE) Platform, Riyadh, Saudi Arabia

**Keywords:** Coronavirus disease, COVID19, Critically ill, Coinfection, Outcomes, Mortality, MV duration, Respiratory viruses

## Abstract

**Background:**

Previous studies have shown that non-critically ill COVID-19 patients co-infected with other respiratory viruses have poor clinical outcomes. However, limited studies focused on this co-infections in critically ill patients. This study aims to evaluate the clinical outcomes of critically ill patients infected with COVID-19 and co-infected by other respiratory viruses.

**Methods:**

A multicenter retrospective cohort study was conducted for all adult patients with COVID-19 who were hospitalized in the ICUs between March, 2020 and July, 2021. Eligible patients were sub-categorized into two groups based on simultaneous co-infection with other respiratory viruses throughout their ICU stay. Influenza A or B, Human Adenovirus (AdV), Human Coronavirus (i.e., 229E, HKU1, NL63, or OC43), Human Metapneumovirus, Human Rhinovirus/Enterovirus, Middle East Respiratory Syndrome Coronavirus (MERS-CoV), Parainfluenza virus, and Respiratory Syncytial Virus (RSV) were among the respiratory viral infections screened. Patients were followed until discharge from the hospital or in-hospital death.

**Results:**

A total of 836 patients were included in the final analysis. Eleven patients (1.3%) were infected concomitantly with other respiratory viruses. Rhinovirus/Enterovirus (38.5%) was the most commonly reported co-infection. No difference was observed between the two groups regarding the 30-day mortality (HR 0.39, 95% CI 0.13, 1.20; *p* = 0.10). The in-hospital mortality was significantly lower among co-infected patients with other respiratory viruses compared with patients who were infected with COVID-19 alone (HR 0.32 95% CI 0.10, 0.97; *p* = 0.04). Patients concomitantly infected with other respiratory viruses had longer median mechanical ventilation (MV) duration and hospital length of stay (LOS).

**Conclusion:**

Critically ill patients with COVID-19 who were concomitantly infected with other respiratory viruses had comparable 30-day mortality to those not concomitantly infected. Further proactive testing and care may be required in the case of co-infection with respiratory viruses and COVID-19. The results of our study need to be confirmed by larger studies.

**Supplementary Information:**

The online version contains supplementary material available at 10.1186/s12879-023-08010-8.

## Introduction

Coronavirus disease 2019 (COVID-19) is a highly contagious infection caused by severe acute respiratory syndrome coronavirus 2 (SARS-CoV-2) [1]. COVID-19 is characterized by respiratory symptoms, including fever, dyspnea, and cough [1]. Other respiratory viruses such as influenza, parainfluenza, and respiratory syncytial virus (RSV) manifested with signs and symptoms similar to COVID-19. This similarity makes it challenging to differentiate subjectively between the various respiratory viral infections [1, 2]. COVID-19 co-infection with other respiratory viruses has been widely detected and was associated with negative clinical outcomes, and increasing the mortality rate [2–5].

A meta-analysis that included 3834 patients diagnosed with COVID-19 showed that 3% were co-infected with other respiratory viruses; the most prevalent viruses were respiratory syncytial virus (RSV) (16.9%), followed by influenza A (15.5%) [2]. Similarly, another meta-analysis that included 10,484 patients showed that 4% were co-infected with other respiratory viral infections, and RSV was the most commonly detected virus [3]. Furthermore, one of the largest cohort studies, including 212,466 patients with COVID-19 and respiratory viral co-infection, detected viral co-infection in 583 patients (8.4%), where influenza viruses, RSV, followed by adenoviruses were the most common viral co-infections [4]. In Saudi Arabia, a small study included 48 patients diagnosed with COVID-19, 34 patients (70.8%) were co-infected with either bacterial or viral respiratory infections [5]. The most commonly detected virus was influenza A (n = 17, 36%), followed by adenovirus, which was detected in ten patients [5].

The co-infection with influenza viruses was significantly associated with a higher risk of requiring invasive mechanical ventilation than patients with COVID-19 mono-infection (*p* = 0.0001). In addition, COVID-19 co-infection with adenoviruses and influenza viruses was associated with higher odds of death (OR = 1.53 and 2.35, respectively) [4]. A previous retrospective cohort study in Saudi Arabia reported a mortality rate of 19% in patients with COVID-19 and respiratory co-infections [5]. Viral co-infection was associated with higher mortality than bacterial co-infection [5]. That study evaluated the clinical outcomes of COVID-19 co-infection, focusing on influenza, and included both intensive care unit (ICU) and non-ICU patients [5].

Several studies reported poor clinical outcomes and increased mortality among patients with COVID-19 who had co-infections [4–6]. However, few were focused on critically ill patients and multiple respiratory viral co-infections [7]. Therefore, this study aims to investigate the clinical outcomes of COVID-19 critically ill patients co-infected by other respiratory viruses (e.g., influenza A or B, RSV, human adenovirus (AdV), human coronavirus, human metapneumovirus, human rhinovirus/enterovirus, middle east respiratory syndrome coronavirus, parainfluenza virus).

## Methods

### Study design

A multicenter retrospective cohort study retrieved the data between March 1, 2020, and July 31, 2021. The study included severely ill COVID-19 adult patients who were admitted to intensive care units (ICUs) during the mentioned period. Respiratory viral infections including SARS-CoV-2, Influenza A or B, Human Adenovirus (AdV), Human Coronavirus (i.e., 229E, HKU1, NL63, or OC43), Human Metapneumovirus, Human Rhinovirus/Enterovirus, Middle East Respiratory Syndrome Coronavirus (MERS-CoV), Parainfluenza virus, and Respiratory Syncytial Virus (RSV). All were screened and detected using Polymerase Chain Reaction (PCR). The patients who met the eligibility criteria were subcategorized into two sub-cohorts based on simultaneous co-infection with SARS-CoV-2 and other respiratory viruses throughout their ICU stay. The follow-up period was continued until the patient’s discharge or if the patient died during hospitalization. The study was performed in adherence to relevant regulations and standards.

### Study participants

All adult critically ill patients (aged ≥ 18 years) with positive SARS-CoV-2 admitted to the ICUs were screened for eligibility. Patients were excluded if the ICU length of stay (LOS) was less than one day, died within the first 24 h of ICU admission, had unknown respiratory viral status (other than COVID-19), or were labeled as do-not-resuscitate (DNR) (or no code) status within 24 hours of ICU admission.

### Study setting

Secondary and tertiary hospitals in different geographical regions within Saudi Arabia were selected and included for this multicenter study. The primary site was King Abdulaziz Medical City (Riyadh), an academic tertiary hospital. In addition to geographical distribution, the center choice was based on medical records availability and the hospitals’ readiness to participate in this national project records. [23].

### Data collection

The Research Electronic Data Capture (REDCap®) software hosted by King Abdullah International Medical Research Center (KAIMRC) was used for data collection and handelling. We gathered data on patients' age, gender, comorbidities, vital signs, laboratory results, severity scores (SOFA, APACHE II, and multiple organ dysfunction scores), respiratory viral infection status (e.g., RSV, influenza A or B), utilization of prone positioning, mechanical ventilation (MV) within 24 h of ICU admission. A renal profile, acute kidney injury (AKI), liver function tests (LFTs), coagulation profile (i.e., INR, aPTT, fibrinogen, D-dimer), and other markers (Procalcitonin, Ferritin, and creatine phosphokinase (CPK)) were also retrieved within 24 h of ICU admission. For the patients who were eligible, the usage of several drugs was documented including corticosteroids, tocilizumab, and pharmacological thrombi-prophylaxis was documented.

### Outcomes

The in-hospital mortality was considered as the primary endpoint. On the other hand, secondary endpoints were 30-day mortality, the duration of MV, ICU/hospital LOS, complications during ICU stay (AKI, liver injury, new-onset atrial fibrillation, thrombosis, pneumonia (hospital/ventilator-acquired), and secondary fungal infection).

### Statistical analysis

As applicable, we reported continuous variables as means and standard deviations (SD), or medians with lower and upper quartiles (Q1, Q3), and categorical variables as frequencies with percentages. Shapiro–Wilk test was used to evaluate continuous variables, and graphical representations (i.e., histograms, Q-Q plots) were created.

The baseline characteristics of both groups were compared utilizing Chi-square or Fisher's exact test for categorical variables. While normally and non-normally distributed continuous variables were compared using the student t-test and Mann–Whitney U test, respectively.

The 30-day and in-hospital mortality were both analyzed using Multivariable Cox proportional hazards regression analyses. Before fitting the Cox model, the proportionality assumptions was evaluated. By generating a log(-log) plot and inspecting the relationship between scaled Schoenfeld residuals and rank-ordered time, a visual assessment of the assumption was carried out.

For the remaining outcomes, multivariable and negative binomial regression analyses were conducted as applicable. Propensity score was included in the regression analysis as one of the model's factors. The estimates, hazard ratio (HR), and odds ratios (OR) with 95% confidence intervals (CI) were reported as applicable. Model fit was evaluated using the Hosmer–Lemeshow goodness-of-fit test. As the cohort of patients in our study was not derived from random selection, therefore no imputation was made for unavailable data. We uitilized SAS version 9.4 for all statistical analyses and a* P* value of < 0.05 was considered statistically significant.

Patients with COVID-19 who were concomitantly infected with another respiratory viruses (active group) were matched to patients who were only infected with COVID-19 (control group) using propensity score matching procedure (Proc PS match) (SAS, Cary, NC) with a ratio of 3:1 based on patient's age, cancer as comorbidity, acute kidney injury, the requirement for MV within 24 h of ICU admission. Patients were matched only if the difference in the logits of the propensity scores for pairs of patients from the two groups was ≤ 0.5 times the pooled estimate of the standard deviation (SD). A greedy nearest neighbor matching method was used in which one patient who was infected with another respiratory viruses (active) matched with three patients who did not (control), which eventually produced the smallest within-pair difference among all available pairs with treated patients.

## Results

**Fig. 1:**
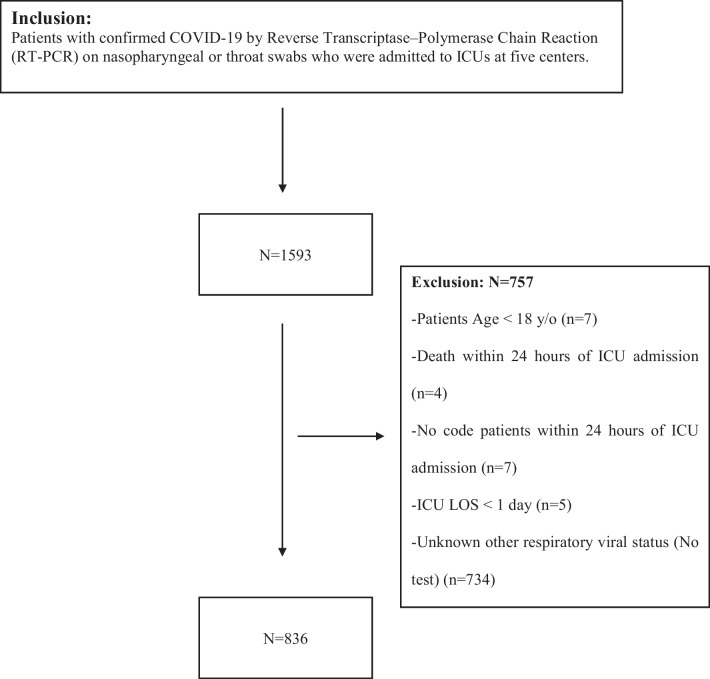
Flow diagram showing patients recruited with COVID-19. *COVID-19*  coronavirus disease, *ICU*  intensive care unit, *LOS*  length of stay

During the study period, 1593 critically ill patients with COVID-19 were screened, and 836 patients were included (Fig. 1). Of the included patients, forty-four were matched using PS with a ratio of 3:1 ratio according to the selected criteria. Among the eligible patients, eleven were infected concomitantly with other respiratory viruses. In patients who were infected concomitantly with other respiratory viruses, Human Rhinovirus/Enterovirus (38.5%) was the most common, followed by Human Adenovirus (AdV) at 23.1%, RSV at 15.4%, and Human Metapneumovirus 7.7% (Table 1).

**Table 1:** Other respiratory viruses concomitant with COVID-19

Other respiratory viruses	Number (%)
Human Adenovirus (AdV)	3 (23.1)
Human Coronavirus (i.e., 229E, HKU1, NL63, or OC43)	0 (0)
Human Metapneumovirus	1 (7.7)
Human Rhinovirus/Enterovirus	5 (38.5)
Middle East Respiratory Syndrome Coronavirus (MeRS cOv)	0 (0)
Parainfluenza virus (i.e., 1,2,3, or 4)	0 (0)
Respiratory Syncytial Virus (RSV)	2 (15.4)
Influenza A or B	0 (0)

### Clinical characteristics of study participants

Most patients were male (62.8%) with a mean age of 62.6 ± 14.53. Hypertension (59.2%) was the most common comorbidity, followed by diabetes mellitus (59.1%), dyslipidemia (25.1%), and chronic kidney disease (11.4%). There was a notable difference in baseline variables between the two groups before PS matching. All patients infected concomitantly with other respiratory viruses required MV within 24 h of ICU admission. Moreover, clinical and laboratory baseline characteristics within 24 h of ICU admission, such as serum creatinine, ferritin, and blood glucose levels were significantly different between the two groups (Additional file 1: Table S1). However, most of the demographic and clinical characteristics of the two groups were generally well balanced after PS matching based on the patient's age, cancer as comorbidity, acute kidney injury, and the need for MV within 24 h of ICU admission. Furthermore, the two groups' use of tocilizumab, methylprednisolone, and dexamethasone was similar within 24 h of ICU admission (Additional file 1: Table S1).

### 30-day and in-hospital mortality

The 30-day mortality in the crude analysis was 73.1% in the COVID-19 mono-infection group compared with 44.4% in patients who were co-infected with other respiratory viruses, which was not statistically significant between the two groups at cox regression analysis (HR 0.39, 95% CI 0.13, 1.20; *p* = 0.10). On the other hand, in-hospital mortality was significantly lower in patients concomitantly infected with other respiratory viruses compared to the COVID-19 mono-infection (HR 0.32 95% CI 0.10, 0.97; *p* = 0.04) (Table 1).

### Mechanical ventilation duration and length of stay

COVID-19 patients co-infected with other respiratory viruses had a statistically significant longer median MV duration than those infected with COVID-19 alone (23.0 versus 12 days: *p* = 0.01) in the crude analysis as well in regression analysis (beta coefficient: 0.55 (95% CI 0.04,1.06), *p* = 0.03)). Furthermore, patients infected concomitantly with other respiratory viruses have a longer hospital LOS when compared to the COVID-19 mono-infection group (beta coefficient: 0.52 (95% CI 0.05, 1.00); *p* = 0.03)). Additionally, the two groups had significant differences in the ICU's LOS in the crude analysis (23.0 vs. 16.5 days; p-value = 0.04). However, this difference did not reach statistical significance in regression analysis (beta coefficient: 0.34 (95% CI (− 0.02, 0.69), *p* = 0.06)) (Table 2).

**Table 2:** Clinical outcomes after propensity score (PS) matching

Outcomes	COVID-19 alone	COVID-19 co-infected with other respiratory viruses	P-value	Hazard ratio (HR) (95%CI)	P-value $
30-day mortality, n (%)∆	19 (73.1)	4 (44.4)	0.12**	0.39 (0.13, 1.20)	0.10
In-hospital mortality, n (%)∆	20 (76.9)	4 (44.4)	0.07**	0.32 (0.10, 0.97)	0.04

				Beta coefficient (Estimates) (95%CI)	P-value $*
MV duration (days), median (Q1,Q3) ∆	12.0 (4.00, 20.00)	23.0 (18.00, 35.00)	0.01^	0.55 (0.04,1.06)	0.03
ICU Length of stay (days), median (Q1, Q3) ∆	16.5 (11.50, 21.50)	23.0 (15.00, 40.00)	0.04^	0.34 (− 0.02, 0.69)	0.06
Hospital length of stay (days), median (Q1, Q3) ∆	22.5 (14.00, 32.50)	31.0 (23.00, 53.00)	0.05^	0.52 (0.05, 1.00)	0.03

∆The denominator of the percentage is the total number of patients

^ Wilcoxon rank sum test is used to calculate the P-value

**Fisher Exact test is used to calculate the P-value

^$^Cox proportional hazards regression analysis used to calculate HR and p-value

^$*^Generalized linear model is used to calculate estimates and p-value

### Complications during ICU stay

Patients with COVID-19 co-infected with other respiratory viruses had a higher odd of all thrombosis cases compared to the COVID-19 mono-infection group, but it was not statistically significant (OR 3.45 (95% CI 0.18,66.95; *p* = 0.41). Other outcomes during ICU stay, such as new-onset atrial fibrillation, AKI, liver injury, hospital/ventilator-acquired pneumonia, and secondary fungal infection, did not differ between the two groups (Table 3).

**Table 3:** Complications during ICU stay

Outcomes	COVID-19	COVID-19 concomitant with other respiratory viral infection	P-value	Odds ratio (OR) (95%CI)	P-value $
New onset A fib., n (%)∆	9 (27.3)	1 (9.1)	0.21**	0.26 (0.03,2.37)	0.23
Acute kidney injury, n(%)∆	17 (51.5)	4 (36.4)	0.38^^	0.52 (0.12,2.19)	0.37
Liver injury, n(%)∆	4 (12.1)	1 (9.1)	0.78**	0.73 (0.07,7.29)	0.79
All thrombosis cases, n(%)∆	1 (3.0)	1 (9.1)	0.40**	3.45 (0.18,66.95)	0.41
Hospital/ventilator acquired pneumonia, n(%)∆	11 (33.3)	3 (27.3)	0.71**	0.37 (0.04,3.41)	0.38
Secondary fungal infection, n(%)∆	5 (15.6)	1 (9.1)	0.59**	0.54 (0.06,5.25)	0.59

∆The denominator of the percentage is the total number of patients

**^^**Chi-square test is used to calculate the P-value

^**^Fisher Exact test is used to calculate the P-value

^$^Logistic regression is used to calculate the OR and p-value

## Discussion

In this multicenter cohort study, we aimed to evaluate the clinical outcomes among critically ill patients with COVID-19 concomitantly infected with another respiratory viruses. The occurrence of 30-day mortality was numerically higher among patients with COVID-19 mono-infection than patients with co-infections but was no statistically significant difference. In contrast, in-hospital mortality was significantly lower in patients co-infected with other respiratory viruses compared to the COVID-19 mono-infection.

Several studies highlighted concomitant infections with other respiratory pathogens that can exacerbate COVID-19 complications and increase mortality [8, 9]. The concurrent infection with other respiratory viruses among critically ill patients with COVID-19 is associated with a lower in-hospital mortality rate compared to COVID-19-infected patients alone. The reduction in the in-hospital mortality in the concomitant infected group compared to the COVID-19 group was statistically significant. However, the 30 days-hospital mortality was lower in the concomitant infected group compared to the COVID-19 group but was not statistically significant. The early use of systemic corticosteroids and tocilizumab was similar between the groups. The comparable rate of using these immunosuppressants and immuno-modulator agents might explain the insignificant difference in the 30 days mortality rate between the two groups in our study.

On the contrary, an updated Cochrane systematic review and meta-analysis suggested that using corticosteroids in viral infections has a threefold increase in mortality and aggravation of inflammation. Hence, viral replication is at its highest and is potentially detrimental by prolonging the duration of viral infections due to delaying in viral clearance, allowing for the accumulation of inflammation-related organ damage [10]. Additionally, it is known that administering systemic corticosteroids has potential adverse outcomes, such as prolonged viral shedding duration in COVID-19 patients. However, the impact of viral shedding on COVID-19 patients' mortality is unclear [11].

Contrary to our findings, a previous study conducted in Saudi Arabia showed a higher mortality rate in ICU patients with COVID-19 and respiratory co-infections than in non-ICU patients with COVID-19 and co-infections [5]. Death was reported in 5 out of 9 patients who were co-infected with the influenza virus [5]. However, this study only included 14 ICU patients with COVID-19 and was conducted at a time when the proven mortality benefits of some therapies were questionable [5]. In addition, a cohort study found that co-infection with respiratory pathogens may increase the mortality rate among critically ill patients with COVID-19 [12]. That study looked at bacterial and fungal co-infection but not viral co-infection [12].

Nevertheless, the cohort in our study had respiratory viral co-infections, and the variation in mortality rate might be specific to the co-infected pathogen. In addition, fungal co-infections may lead to worse clinical outcomes in patients with COVID-19 in comparison to viral and bacterial co-infections [2]. Compared to bacterial and fungal co-infections, viral co-infections occurred relatively rarely, affecting approximately 3% of patients. Moreover, none of the critically ill patients with COVID-19 included in our study was co-infected with influenza. The low rate of influenza co-infection in patients with COVID-19 has also been reported in a previous systematic review and meta-analysis that evaluated the prevalence of influenza co-infection [22]. These low rates may be attributed to the low testing rate of other viral co-infections in patients with COVID-19. Nonetheless, due to the remarkable advances made in the diagnosis and treatment, influenza is significantly less associated with mortality.

In our study, patients with co-infections were more likely to require MV than patients with COVID-19 mono-infection and required longer MV duration. Hence, the co-infected group survived with a lower mortality rate. Similarly, a retrospective cohort study investigated the co-infection and its association with 30 days mortality and outcomes [13]. They included COVID-19-positive patients who were also tested for other respiratory viruses and aimed to determine if co-infection with COVID-19 was associated with more severe presentation and outcomes [13]. The study demonstrated that the need for MV or death within 30 days was higher in the COVID-19 group, with 37% vs. 21.4% in the co-infected group (*p* = 0.24) [13]. The data does not, however, accurately reflect the number of patients that required MV or died within 30 days of being admitted to the hospital since that retrospective study did not separate the outcomes [13].

The ICU and MV durations were significantly higher in the group with co-infection. In other words, patients who were critically ill in this study showed lower mortality rates and more prolonged need for ICU stay and required a longer duration of MV compared to patients with COVID-19 mono-infection. Regarding the LOS, our study found that patients with COVID-19 and co-infection required longer hospital and ICU stay than the COVID-19 group alone (hospital 31%, ICU 23% vs. hospital 16.5%, ICU 22.5%), respectively. On the contrary, a retrospective cohort study divided the population into two groups: the COVID-19 cohort with a negative respiratory pathogen panel (RPP); and the COVID-19 co-infected cohort who tested positive for at least one viral target on the RPP [13]. That study concluded that co-infected patients had shorter LOS with an overall 6.9 vs. 16.2 days (*p* = 0.80) [13]. Another retrospective study included patients admitted to the ICU with co-infection and COVID-19 and found that bacterial co-infection increased the length of ICU hospitalization [14]. However, that study did not include co-infection with other viral respiratory pathogens [14]. The presence of other comorbidities might explain the difference in LOS between the studies.

Inflammation status, specifically high ferritin levels, can serve as a biomarker to predict outcomes and severity of COVID-19 infection [15]. A meta-analysis associated high ferritin levels with severe COVID-19 infections compared to non-severe infections [15]. Also, clinical outcomes such as death and the need for MV was seen more in patients with high ferritin level [15]. However, the previously mentioned meta-analysis had high heterogeneity [15]. Both of the study’s groups were critically ill, which is why both of them had elevated ferritin levels. The co-infected group had a higher ferritin level, although it was not statistically significant.

Hypertension and diabetes were the most common comorbidities found in our study in both groups, which is aligned with another retrospective cohort study [16]. The rate of comorbidities was higher in the COVID-19 mono-infection group, which might explain the increased mortality rate compared to the co-infected group. Additionally, the co-infected group had a lower mortality rate, which might be translated to longer hospital or ICU length of stay as these patients have survived but might stay longer for medical support and treatments.

Several studies suggested that viral co-infection generally leads to worse clinical outcomes [17–19]. Patients with respiratory co-infections have been observed to have more complications. The following complications were listed in a meta-analysis: renal, hepatobiliary, hematological, neurological, cardiac, and complications with the neurological system [20]. Recent studies have demonstrated that pulmonary co-infection caused by other agents is not uncommon in patients with COVID-19 [21]. The COVID-19 mono-infection group in our study had higher rates of thrombosis at baseline and before the PS matching than the co-infected group; however, this complication was not statistically significant in both groups after the propensity score matching (*p* = 0.04). We could not compare our findings with other published studies due to the lack of available data in this regard.

We believe that our multicenter cohort study is the first study to evaluate the clinical outcomes among critically ill patients with COVID-19 mono-infection compared with those with COVID-19 and viral co-infection. Through this study, we were able to compare the clinical outcomes between patients with COVID-19 mono-infection and those who also had co-infections with respiratory viruses. Nevertheless, we also have to acknowledge some limitations to our study. First, the study's retrospective nature makes it difficult to determine whether our findings can be applied to all COVID-19 and viral co-infected patients, especially (MeRS CoV, influenza, and parainfluenza co-infections). Second, we could not report the exact rate of clinical outcomes in each co-infection with a viral pathogen type. Also, vaccination status and COVID-19 variants were not reported in our study, which can influence different clinical outcomes.

## Conclusion

In this cohort study, critically ill patients with COVID-19 who were concomitantly infected with other respiratory viruses had a comparable 30-day mortality compared to those not concomitantly infected. Further proactive testing and care may be required in the case of co-infection with respiratory viruses and COVID-19. The results of our study need to be confirmed by larger studies.

## Supplementary Information

**Additional file 1: Table S1.** Baseline characteristic of critically ill patients before and after propensity score matching.

## Data Availability

The datasets generated during and analyzed during the current study are not publicly available due to privacy and ethical restrictions but are available from the corresponding author on reasonable request.
