# Correction: The clinical outcomes of COVID-19 critically ill patients co-infected with other respiratory viruses: a multicenter, cohort study

**DOI:** 10.1186/s12879-023-08072-8

**Published:** 2023-02-22

**Authors:** Khalid Al Sulaiman, Ohoud Aljuhani, Hisham A. Badreldin, Ghazwa B. Korayem, Abeer A. Alenazi, Ahlam H. Alharbi, Albandari Alghamdi, Alaa Alhubaishi, Ali F. Altebainawi, Mohammad Bosaeed, Rand Alotaibi, Ahad Alawad, Nirvana Alnajjar, Khalid Bin Saleh, Walaa A. Sait, Samiah Alsohimi, Meshari M. Alanizy, Sarah A. Almuqbil, Ibrahim Al Sulaihim, Ramesh Vishwakarma, Mai Alalawi, Fatimah Alhassan, Suliman Alghnam

**Affiliations:** 1 Pharmaceutical Care Department, King Abdulaziz Medical City (KAMC)-Ministry of National Guard Health Afairs (MNGHA), Riyadh, Saudi Arabia; 2 College of Pharmacy, King Saud Bin Abdulaziz University for Health Sciences, P.O. Box 22490, Riyadh, 11426 Saudi Arabia; 3 King Abdullah International Medical Research Center, Riyadh, Saudi Arabia; 4 Norwich Medical School, University of East Anglia, Norwich, UK; 5 Department of Pharmacy Practice, Faculty of Pharmacy, King Abdulaziz University, Jeddah, Saudi Arabia; 6 Pharmaceutical Care Services, King Khalid Hospital, Hail Health Cluster, Hail, Saudi Arabia; 7 Department of Pharmacy Practice, College of Pharmacy, Princess Nourah Bint Abdulrahman University, P.O. Box 84428, Riyadh, 11671 Saudi Arabia; 8 Pharmaceutical Care Department, Prince Sultan Military Medical City, Riyadh, Saudi Arabia; 9 Department of Pharmaceutical Sciences, Fakeeh College for Medical Sciences, Jeddah, Saudi Arabia; 10 Population Health Section, King Abdullah International Medical Research Center, Riyadh, Saudi Arabia; 11 Infectious Disease Care Department, King Abdulaziz Medical City, Riyadh, Saudi Arabia; 12 College of Medicine, King Saud Bin Abdulaziz University for Health Sciences, Riyadh, Saudi Arabia; 13 College of Pharmacy, Qassim University, Qassim, Saudi Arabia; 14 Pharmaceutical Care Department, King Abdulaziz Medical City, Jeddah, Saudi Arabia; 15 Pharmaceutical Care Department, King Abdulaziz University Hospital, Jeddah, Saudi Arabia; 16 Central Security Hospital, Riyadh, Saudi Arabia; 17 Pharmaceutical Care Department, King Fahad Armed Forces Hospital, Jeddah, Saudi Arabia; 18 Saudi Critical Care Pharmacy Research (SCAPE) Platform, Riyadh, Saudi Arabia

**Correction: BMC Infectious Diseases (2023) 23:75** 10.1186/s12879-023-08010-8

Following publication of the original article [1], the authors identified an error in the affiliation of author Ahlam H. Alharbi.

- The incorrect author affiliation is: Ahlam H. Alharbi^13^
- The correct author affiliation is: Ahlam H. Alharbi^8^

The author group has been updated above and the original article [1] has been corrected.
