# Supplementary material for: The clinical outcomes of COVID-19 critically ill patients co-infected with other respiratory viruses: a multicenter, cohort study
Source: BMC Infect Dis. 2023 Feb 6;23:75. doi: 10.1186/s12879-023-08010-8 (PMC9901824; doi:10.1186/s12879-023-08010-8)
Supplement: Supplementary file 1 — Additional file 1: Table S1. Baseline characteristic of critically ill patients before and after propensity score matching. [file 12879_2023_8010_MOESM1_ESM.docx]

**Additional file 1:Table S1. Baseline characteristic before and after propensity score matching**

|  | **Before propensity score (PS)** | | | | **After propensity score (PS)** | | | |
| --- | --- | --- | --- | --- | --- | --- | --- | --- |
|  | **Overall (N=836)** | **COVID-19 alone (N=825)** | **COVID-19+ Other Viruses (N=11)** | **P-value** | **Overall (N=44)** | **COVID-19 alone (N=33)** | **COVID-19+ Other Viruses (N=11)** | **P-value** |
| **Age (Years), Mean (SD)** | 62.6 (14.53) | 62.5 (14.51) | 69.0 (15.87) | 0.1351^ | 71.2 (15.77) | 71.9 (15.92) | 69.0 (15.87) | 0.6329^ |
| **Gender – Male, n (%)** | 518 ( 62.8 ) | 511 ( 62.8 ) | 7 ( 63.6 ) | 0.9533** | 28 ( 63.6 ) | 21 ( 63.6 ) | 7 ( 63.6 ) | >0.9999** |
| **Weight (kg), Mean (SD)** | 82.5 (20.50) | 82.6 (20.54) | 75.0 (16.27) | 0.2644^ | 77.4 (14.65) | 78.2 (14.26) | 75.0 (16.27) | 0.5419* |
| **APACHE II score, Median (Q1,Q3)** | 12.0 (8.00, 19.00) | 12.0 (8.00, 19.00) | 13.0 (12.00, 20.00) | 0.4437^ | 13.0 (11.00, 18.00) | 14.0 (11.00, 18.00) | 13.0 (12.00, 20.00) | 0.7721* |
| **SOFA score, Median (Q1,Q3)** | 4.0 (2.00, 7.00) | 4.0 (2.00, 7.00) | 4.0 (3.00, 7.00) | 0.8521^ | 4.5 (3.00, 6.00) | 5.0 (3.00, 7.00) | 3.0 (2.00, 5.00) | 0.1246* |
| **Multiple Organ Dysfunction Score, Median (Q1,Q3)** | 5.0 (4.00, 7.00) | 5.0 (4.00, 7.00) | 6.5 (4.50, 7.00) | 0.6573^ | 5.0 (4.00, 7.00) | 5.0 (4.00, 6.00) | 6.5 (4.50, 7.00) | 0.3998^ |
| **Early use of Dexamethasone within 24 hours, n (%)** | 507 ( 60.6 ) | 500 ( 60.6 ) | 7 ( 63.6 ) | 0.8381** | 28 ( 63.6 ) | 21 ( 63.6 ) | 7 ( 63.6 ) | >0.9999** |
| **Early use of Tocilizumab within 24 hours, n (%)** | 174 ( 20.8 ) | 172 ( 20.8 ) | 2 ( 18.2 ) | 0.8287** | 6 ( 13.6 ) | 4 ( 12.1 ) | 2 ( 18.2 ) | 0.6120** |
| **Serum creatinine (mmol/L) baseline, Median (Q1,Q3)** | 84.0 (68.00, 122.00) | 84.0 (68.00, 123.00) | 66.0 (66.00, 78.00) | 0.0396^ | 76.5 (66.00, 107.50) | 82.0 (68.00, 110.00) | 66.0 (66.00, 78.00) | 0.0926^ |
| **Blood Urea nitrogen (BUN) baseline (mmol/L), Median (Q1,Q3)** | 6.8 (4.70, 11.50) | 6.8 (4.70, 11.45) | 7.7 (4.90, 13.00) | 0.9558^ | 7.5 (4.90, 13.00) | 7.5 (5.00, 12.10) | 7.7 (4.90, 13.00) | 0.8022^ |
| **Acute Kidney Injury (AKI) Within 24 hours of ICU admission, n (%)** | 242 ( 29.3 ) | 241 ( 29.6 ) | 1 ( 9.1 ) | 0.1382** | 4 ( 9.1 ) | 3 ( 9.1 ) | 1 ( 9.1 ) | >0.9999** |
| **Mechanical Ventilation within 24 hours of ICU admission, n (%)** | 599 ( 72.3 ) | 588 ( 71.9 ) | 11 (100.0 ) | 0.0386** | 44 (100.0 ) | 33 (100.0 ) | 11 (100.0 ) | NA |
| **A-A Gradient baseline , Median (Q1,Q3)** | 419.6 (258.91, 557.42) | 418.9 (258.78, 560.57) | 471.5 (277.93, 497.34) | 0.8596^ | 432.6 (328.84, 487.11) | 423.6 (334.02, 483.07) | 471.5 (277.93, 497.34) | 0.7924* |
| **Oxygenation Index (OI) baseline, Median (Q1,Q3)** | 17.3 (10.04, 27.27) | 17.3 (10.04, 27.27) | 21.7 (14.79, 83.41) | 0.4420^ | 19.4 (8.16, 24.28) | 19.3 (8.16, 24.28) | 21.7 (14.79, 83.41) | 0.5334^ |
| **Inotropes/vasopressors use within 24 hours of admission), n(%)** | 214 ( 26.0 ) | 210 ( 25.9 ) | 4 ( 36.4 ) | 0.4303** | 14 ( 31.8 ) | 10 ( 30.3 ) | 4 ( 36.4 ) | 0.7086** |
| **Lactic acid baseline (mmol/l), Median (Q1,Q3)** | 1.7 (1.29, 2.44) | 1.7 (1.29, 2.44) | 2.0 (1.44, 2.13) | 0.7372^ | 1.9 (1.28, 2.13) | 1.9 (1.21, 2.10) | 2.0 (1.44, 2.13) | 0.3326* |
| **Platelets count baseline (10^9^/l), Median (Q1,Q3)** | 243.0 (188.00, 316.00) | 243.0 (188.00, 316.00) | 222.0 (167.00, 348.00) | 0.8525^ | 240.0 (193.00, 336.00) | 245.0 (193.00, 335.00) | 222.0 (167.00, 348.00) | 0.9224^ |
| **Total WBC baseline 10^9^/l), Median (Q1,Q3)** | 9.1 (6.24, 12.50) | 9.1 (6.30, 12.55) | 6.3 (5.36, 11.00) | 0.1539^ | 8.8 (5.54, 12.25) | 9.0 (6.53, 12.50) | 6.3 (5.36, 11.00) | 0.2447* |
| **International normalized ratio (INR), Median (Q1,Q3)** | 1.1 (1.00, 1.17) | 1.1 (1.00, 1.17) | 1.0 (0.96, 1.05) | 0.0834^ | 1.1 (1.00, 1.12) | 1.1 (1.00, 1.14) | 1.0 (0.96, 1.05) | 0.2835^ |
| **Activated partial thromboplastin time (aPTT) baseline (Seconds), Median (Q1,Q3)** | 29.5 (26.40, 33.00) | 29.5 (26.40, 33.20) | 27.8 (24.80, 32.00) | 0.2056^ | 28.6 (25.85, 32.75) | 29.1 (26.00, 34.00) | 27.8 (24.80, 32.00) | 0.3567^ |
| **Total bilirubin baseline (umol/l), Median (Q1,Q3)** | 10.0 (7.20, 15.40) | 10.1 (7.20, 15.40) | 9.0 (7.50, 17.90) | 0.8927^ | 10.5 (7.50, 19.00) | 13.3 (7.90, 19.30) | 9.0 (7.50, 17.90) | 0.4972^ |
| **Alanine transaminase (ALT) baseline (U/L), Median (Q1,Q3)** | 35.0 (23.00, 56.00) | 35.0 (23.00, 56.00) | 37.5 (26.00, 68.00) | 0.5506^ | 37.5 (26.00, 54.00) | 39.0 (24.00, 48.00) | 37.5 (26.00, 68.00) | 0.5544^ |
| **Aspartate transaminase (AST) baseline (U/L), Median (Q1,Q3)** | 49.5 (33.00, 73.00) | 50.0 (33.00, 73.00) | 45.0 (31.00, 55.00) | 0.5433^ | 55.0 (37.00, 75.00) | 58.5 (37.50, 78.00) | 45.0 (31.00, 55.00) | 0.2145^ |
| **Albumin baseline (gm/l), Median (Q1,Q3)** | 33.0 (29.00, 36.00) | 33.0 (29.00, 36.00) | 31.0 (31.00, 38.00) | 0.9201^ | 33.0 (30.00, 37.00) | 33.0 (29.00, 37.00) | 31.0 (31.00, 38.00) | 0.8204* |
| **C-reactive protein (CRP) baseline (mg/l), Median (Q1,Q3)** | 79.3 (26.00, 162.50) | 79.6 (26.00, 164.00) | 75.0 (38.47, 110.00) | 0.7392^ | 81.9 (40.50, 158.00) | 87.7 (46.00, 177.00) | 75.0 (38.47, 110.00) | 0.5116^ |
| **Fibrinogen Level baseline (gm/l), Median (Q1,Q3)** | 5.4 (3.90, 7.05) | 5.4 (3.90, 7.09) | 5.0 (4.36, 6.60) | 0.8095^ | 5.3 (4.36, 6.07) | 5.7 (3.37, 6.07) | 5.0 (4.36, 6.60) | 0.8726* |
| **D-dimer Level baseline (mg/l), Median (Q1,Q3)** | 1.2 (0.71, 3.00) | 1.2 (0.71, 2.98) | 2.5 (1.09, 6.07) | 0.2828^ | 1.1 (0.66, 3.45) | 1.1 (0.66, 2.48) | 2.5 (1.09, 6.07) | 0.2935^ |
| **Ferritin Level baseline (ug/l), Median (Q1,Q3)** | 712.3 (384.15, 1747.55) | 698.0 (384.00, 1635.60) | 2335.4 (1071.00, 5364.70) | 0.0398^ | 688.9 (364.00, 2335.40) | 609.3 (357.00, 929.90) | 2335.4 (1071.00, 5364.70) | 0.0770^ |
| **Blood glucose level baseline (mmol/l), Median (Q1,Q3)** | 10.8 (7.50, 15.00) | 10.9 (7.50, 15.10) | 7.2 (5.80, 13.90) | 0.0304^ | 8.0 (6.60, 11.00) | 8.4 (7.00, 11.00) | 7.2 (5.80, 13.90) | 0.4267^ |
| **PaO_2_/FiO_2_ ratio within 24 hours of admission, Median (Q1,Q3)** | 81.6 (60.00, 129.11) | 81.6 (59.95, 129.06) | 76.0 (63.38, 129.82) | 0.9091^ | 79.3 (63.38, 105.45) | 79.3 (61.06, 101.53) | 76.0 (63.38, 129.82) | 0.7790^ |
| **Respiratory rate (Breath Per Minute) baseline, Median (Q1,Q3)** | 28.0 (24.00, 34.00) | 28.0 (24.00, 34.00) | 26.0 (21.00, 33.00) | 0.4928^ | 26.0 (22.00, 30.00) | 26.0 (22.00, 30.00) | 26.0 (21.00, 33.00) | 0.3530* |
| **Highest heart rate (HR) baseline (BPM), Median (Q1,Q3)** | 103.0 (91.00, 114.00) | 103.0 (91.00, 114.00) | 96.0 (87.00, 118.00) | 0.8043^ | 102.0 (94.00, 114.50) | 103.0 (94.00, 114.00) | 96.0 (87.00, 118.00) | 0.6446^ |
| **Lowest MAP baseline (mmHg), Median (Q1,Q3)** | 71.0 (63.00, 81.00) | 71.0 (63.00, 81.00) | 73.0 (60.00, 78.00) | 0.7355* | 70.0 (63.00, 78.00) | 69.0 (63.00, 80.00) | 73.0 (60.00, 78.00) | 0.9055* |
| **Pharmacological DVT prophylaxis use during ICU stay ,n (%)** | 793 ( 95.5 ) | 783 ( 95.5 ) | 10 (100.0 ) | 0.4919** | 41 ( 95.3 ) | 31 ( 93.9 ) | 10 (100.0 ) | 0.4253** |
| High dose of Pharmacological DVT prophylaxis, n(%)≠ | 320 ( 40.5 ) | 317 ( 40.5 ) | 3 ( 33.3 ) | 0.5071** | 7 ( 17.5 ) | 4 ( 12.9 ) | 3 ( 33.3 ) | 0.2087** |
| Standard dose of Pharmacological DVT prophylaxis, n(%)≠ | 402 ( 50.8 ) | 396 ( 50.6 ) | 6 ( 66.7 ) | 0.5071** | 28 ( 70.0 ) | 22 ( 71.0 ) | 6 ( 66.7 ) | 0.2087** |
| Low dose of Pharmacological DVT prophylaxis, n(%)≠ | 69 ( 8.7 ) | 69 ( 8.8 ) | 0 ( 0.0 ) | 0.5071** | 5 ( 12.5 ) | 5 ( 16.1 ) | 0 ( 0.0 ) | 0.2087** |
| **Patient received nephrotoxic drugs/material during ICU stay, n (%)*$** | 736 ( 89.0 ) | 727 ( 89.0 ) | 9 ( 90.0 ) | 0.9187** | 40 ( 93.0 ) | 31 ( 93.9 ) | 9 ( 90.0 ) | 0.6684** |
| **Comorbidity, n (%)** |  |  |  |  |  |  |  |  |
| Atrial fibrillation (A Fib) | 34 ( 4.1 ) | 34 ( 4.1 ) | 0 ( 0.0 ) | 0.4918** | 1 ( 2.3 ) | 1 ( 3.0 ) | 0 ( 0.0 ) | 0.5592** |
| Heart Failure | 58 ( 6.9 ) | 57 ( 6.9 ) | 1 ( 9.1 ) | 0.7772** | 3 ( 6.8 ) | 2 ( 6.1 ) | 1 ( 9.1 ) | 0.7299** |
| Hypertension | 495 ( 59.2 ) | 486 ( 58.9 ) | 9 ( 81.8 ) | 0.1246** | 31 ( 70.5 ) | 22 ( 66.7 ) | 9 ( 81.8 ) | 0.3402** |
| Diabetes Mellitus | 494 ( 59.1 ) | 488 ( 59.2 ) | 6 ( 54.5 ) | 0.7576** | 25 ( 56.8 ) | 19 ( 57.6 ) | 6 ( 54.5 ) | 0.8605** |
| Dyslipidemia | 210 ( 25.1 ) | 207 ( 25.1 ) | 3 ( 27.3 ) | 0.8684** | 14 ( 31.8 ) | 11 ( 33.3 ) | 3 ( 27.3 ) | 0.7086** |
| Ischemic heart disease (IHD) | 75 ( 9.0 ) | 74 ( 9.0 ) | 1 ( 9.1 ) | 0.9888** | 4 ( 9.1 ) | 3 ( 9.1 ) | 1 ( 9.1 ) | >0.9999** |
| Chronic kidney disease (CKD) | 95 ( 11.4 ) | 95 ( 11.5 ) | 0 ( 0.0 ) | 0.2319** | 4 ( 9.1 ) | 4 ( 12.1 ) | 0 ( 0.0 ) | 0.2259** |
| Cancer | 42 ( 5.0 ) | 39 ( 4.7 ) | 3 ( 27.3 ) | 0.0007** | 10 ( 22.7 ) | 7 ( 21.2 ) | 3 ( 27.3 ) | 0.6779** |
| Deep Vein Thrombosis (DVT) | 8 ( 1.0 ) | 8 ( 1.0 ) | 0 ( 0.0 ) | 0.7428** | 1 ( 2.3 ) | 1 ( 3.0 ) | 0 ( 0.0 ) | 0.5592** |
| Pulmonary Embolism (PE) | 5 ( 0.6 ) | 4 ( 0.5 ) | 1 ( 9.1 ) | 0.0002** | 2 ( 4.5 ) | 1 ( 3.0 ) | 1 ( 9.1 ) | 0.4033** |
| Liver disease (any type) | 22 ( 2.6 ) | 22 ( 2.7 ) | 0 ( 0.0 ) | 0.5831** | 0 (0) | 0 (0) | 0 (0) | NA |
| Stroke | 51 ( 6.1 ) | 51 ( 6.2 ) | 0 ( 0.0 ) | 0.3948** | 3 ( 6.8 ) | 3 ( 9.1 ) | 0 ( 0.0 ) | 0.3002** |
| *T Test / ^ Wilcoxon rank sum test is used to calculate the P-value.  ^^ Chi square/ ** Fisher’s Exact teat is used to calculate P-value.  *$ Nephrotoxic medications/ material included IV Vancomycin, Gentamicin, Amikacin, Contrast, Colistin, Furosemide, and/or Sulfamethoxazole/trimethoprim  ≠ Patients who received either Enoxaparin 40 mg daily or UFH 5000 Unit three times daily were grouped under the "standard dose VTE prophylaxis. Any patient who received higher than standard dose but not as treatment dose (Enoxaparin 1mg/kg q12hr or 1.5mg/kg q24hr or UFH infusion) was categorized as receiving "High VTE prophylaxis dose". On the other hand, lower VTE prophylaxis considered for patient who received Enoxaparin <40 mg/day or Unfractionated heparin (UFH ) <5000 Units three times daily/day). | | | | | | | | |
